# National and State-Specific Unit Sales and Prices for Electronic Cigarettes, United States, 2012–2016

**DOI:** 10.5888/pcd15.170555

**Published:** 2018-08-02

**Authors:** Teresa W. Wang, Ellen M. Coats, Doris G. Gammon, Brett R. Loomis, Nicole M. Kuiper, Todd Rogers, Brian A. King

**Affiliations:** 1 Office on Smoking and Health, National Center for Chronic Disease Prevention and Health Promotion, Centers for Disease Control and Prevention, Atlanta, Georgia; 2 Center for Health Policy Science and Tobacco Research, RTI International, Research Triangle Park, North Carolina

## Abstract

**Introduction:**

Few studies have explored patterns of electronic cigarette (e-cigarette) sales and prices by product type over time. We used US retail scanner data to assess national and state-specific trends in e-cigarette unit sales and prices for 4 product types sold from 2012 through 2016.

**Methods:**

Using retail scanner data from the 48 contiguous states and Washington, DC, for convenience stores; supermarkets; mass merchandisers; drug, dollar, and club stores; and military commissaries, we assessed data on monthly unit sales and inflation-adjusted prices by 4 products (rechargeables, disposables, prefilled cartridges, and e-liquids) sold during the 5-year study period. We evaluated national and state trends by using Joinpoint regression (*P* < .05).

**Results:**

From 2012 through 2016, average national monthly unit sales significantly increased for all products, while average monthly prices of rechargeables, disposables, and prefilled cartridges significantly decreased. In 2016, prefilled cartridges had the highest average sales (766 units per 100,000 people) and the lowest average price ($14.36 per unit). By state, average monthly sales significantly increased for at least 1 of 4 e-cigarette products in all 48 states and Washington, DC. However, during the same period, average monthly prices significantly decreased in 39 states for rechargeables, in 31 states for disposables, in 20 states for prefilled cartridges, and in 8 states for e-liquids.

**Conclusion:**

Overall, US e-cigarette unit sales generally increased as product prices decreased. These findings demonstrate the rapidly evolving landscape of US e-cigarette retail marketplace. Ongoing surveillance of e-cigarette unit sales and price is critical for informing and evaluating evidence-based tobacco control strategies.

## Introduction

Electronic cigarettes (e-cigarettes) are a diverse product class of battery-powered devices designed to deliver a combination of nicotine, flavorings, and other additives via an inhaled aerosol (1). Since their entry into the US marketplace in 2007, e-cigarettes have rapidly evolved in product design, marketing, and availability (2,3). In 2014, researchers identified more than 460 e-cigarette brands and 7,700 e-liquid flavors (4). These products are now widely distributed through traditional retail outlets, vape shops, and online retailers (5).

This surge in product availability coincided with increased e-cigarette use, particularly among current and former adult smokers (6). E-cigarette use increased 900% among US high school students from 2011 to 2015, and e-cigarettes surpassed conventional cigarettes as the most commonly used tobacco product among this group (7). The prominent use of e-cigarettes among US youth has been attributed in part to the heavy marketing of these products with youth-resonating themes, as well as the widespread availability of youth-appealing flavors (7,8).

As the tobacco product retail landscape continues to evolve, market surveillance using objective retail sales data can complement self-reported measures to enhance our understanding of consumption patterns and assess the impact of tobacco product regulations (9–13). Proportionate sales of e-cigarettes have been small relative to sales of other tobacco products, especially combustible products. At the end of 2015, cigarette sales were 64 times higher than e-cigarette sales in convenience stores and 73 times higher in “all other outlets combined” (10). However, retail sales data that document the growth in consumption of these products warrants continued monitoring (4,5). To date, few studies have explored e-cigarette sale and price patterns at the national and state levels in the United States (10,13). Furthermore, the extent of more recent e-cigarette sales and price trends has not been examined, and no study has provided a nuanced assessment by product type at both levels. We addressed these gaps by using retail scanner data to assess overall national and state trends in e-cigarette prices and unit sales for 4 product types during the 5-year period from 2012 through 2016.

## Methods

We acquired data on Universal Product Code (UPC) retail sales for e-cigarette products from The Nielsen Company (www.nielsen.com/us/en.html). These data include data on sales from convenience stores (franchise, chain, and independent convenience stores that may or may not sell gasoline) and all other outlets combined (a category that includes mass merchandisers; supermarkets; drug, dollar, and club stores; and military commissaries). Sales are reported in approximately monthly (4-week) aggregates; these period-to-period changes are referred to as monthly changes.

Data were obtained for sales occurring from January 12, 2012, through January 7, 2017. Given that the final 4-week period occurred primarily in 2016, the overall study period is referred to as “2012–2016” hereinafter. We analyzed combined sales at convenience stores and all other outlets combined across the 48 contiguous states and Washington, DC. Sales projections for Alaska and Hawaii were not available from Nielsen and, thus, were not included in our study.

### Measures

The sales data include detailed information on the type of e-cigarette device, brand, flavor, strength, and count per item. Using UPC-specific information, we classified items into 4 mutually exclusive products: 1) rechargeable e-cigarette devices (rechargeables), 2) disposable e-cigarette devices (disposables), 3) disposable cartridges prefilled with e-liquid (prefilled cartridges), and 4) e-liquid bottles for filling reusable cartridges (e-liquids).

Items associated with the descriptor “KIT” or those containing at least one refill, rechargeable battery, and battery charger were classified as rechargeables. Disposables include single-use products that cannot be recharged or refilled. E-cigarette accessories, including lanyards and replacement parts sold without e-liquid, were excluded from our analysis. We reviewed brand websites when we could not determine the product from the information provided in the Nielsen data set (~5% of UPCs).

### Analysis

We calculated sales volume by using standardized unit sales. Within each product category, we standardized unit sales to represent the most common package size for each product type. A standardized unit, henceforth “unit,” equals 1 rechargeable, 1 disposable, 5 prefilled cartridges, or 1 e-liquid bottle. For example, raw unit sales for a UPC indicating a pack of 10 prefilled cartridges were multiplied by 2 to reflect the equivalent number of units sold as a pack of 5. Unit sales were aggregated to produce total monthly sales by product, geography, and time period. We tabulated all national and state unit sales as sales rates of unit sales per 100,000 people (all ages), which was calculated by using respective population estimates from the US Census Bureau (14).

Average prices by product were calculated by using adjusted dollars and nonstandardized units. To generate inflation-adjusted dollar sales, we indexed dollar sales to the 2016 Consumer Price Index from the US Bureau of Labor Statistics (15). Prices were then calculated by using only items sold in the most common package size for each product. For example, only prefilled cartridges that were sold in a pack of 5 were included in price calculations for prefilled cartridges. Although standardizing a pack of 10 prefilled cartridges to represent 2 packs of 5 is appropriate for reflecting sales volume, these sales were excluded from price calculations to avoid the introduction of bias due to discounts associated with buying in bulk.

We evaluated the overall trend from 2012 through 2016 by using Joinpoint models that controlled for autocorrelation to quantify and test the direction and significance (*P* < .05) of the average monthly percentage change (AMPC) (16). Additionally, for 2012 and 2016 we determined an average monthly sales rate and assessed year-to-year percentage change during the study period. Although the national data indicated e-liquid sales began in mid-2013, e-liquid sales did not begin until early 2014 in some states. Therefore, the study period for e-liquid sales includes only periods from 2014 through 2016 that had non-zero sales.

## Results

Nationally, the average monthly e-cigarette sales rate as summed across all product types sold (ie, acknowledging no observable e-liquid sales in 2012) increased by 132%, from 667 units per 100,000 people in 2012 to 1,547 units per 100,000 people in 2016 (Table 1). Unit sales increased by 154% for rechargeables (AMPC = 1.7), 27% for disposables (AMPC = 1.0), 256% for prefilled cartridges (AMPC = 2.4), and 64% for e-liquids (AMPC = 5.9). The average monthly sales rate was highest in 2016 among prefilled cartridges (766 units), followed by disposables (445 units), rechargeables (259 units), and e-liquids (77 units).

**Table 1:** E-Cigarette Unit Sales by Product Type, United States, 2012–2016

State	Rechargeables				Disposables				Prefilled Cartridges				E-Liquids			
	Monthly Unit Sales^a^		Change, %	AMPC^b^	Monthly Unit Sales^a^		Change, %	AMPC^b^	Monthly Unit Sales^a^		Change, %	AMPC^b^	Monthly Unit Sales^a^		Change, %^d^	AMPC^b^
	2012	2016			2012	2016			2012	2016			2014	2016		
**All**	102	259	154	1.7^c^	350	445	27	1.0^c^	215	766	256	2.4^c^	47	77	64	5.9^c^
**Northeast**																
CT	60	103	72	0	266	553	108	1.2	155	453	192	1.1^c^	16	44	175	17.8^c^
MA	59	226	283	1.4	469	746	59	1.2^c^	133	955	618	3.8^c^	52	93	79	2.8
ME	169	485	187	2.2^c^	327	620	90	2.8^c^	273	1,360	398	3.1^c^	42	144	243	7.6^c^
NH	157	622	296	2.3^c^	480	1,381	188	3.0^c^	218	1,569	620	3.9^c^	84	149	77	5.7^c^
NJ	45	320	611	3.3^c^	702	824	17	1.3^c^	169	1,505	791	3.7^c^	50	74	48	11.7^c^
NY	50	234	368	2.5^c^	372	768	106	1.9^c^	120	1,059	783	3.7^c^	45	35	−22	3.6^c^
PA	69	305	342	2.0^c^	349	350	0	0	198	972	391	2.8^c^	47	81	72	8.7^c^
RI	66	226	242	2.1^c^	376	427	14	1.0	208	859	313	2.8^c^	40	120	200	19.4^c^
VT	147	143	−3	0.1	101	460	355	4.7^c^	58	636	997	4.6^c^	12	60	400	11.7^c^
**Midwest**																
IA	36	189	425	3	258	312	21	1.4	90	580	544	3.8^c^	14	34	143	11.8^c^
IL	151	225	49	1.3	288	1,527	430	3.6^c^	239	740	210	2.7^c^	34	110	224	8.0^c^
IN	162	306	89	0.8	219	241	10	0.8	268	770	187	2.1^c^	42	63	50	0.8
KS	65	225	246	2.5^c^	275	183	−33	−0.8	232	475	105	1.6^c^	44	62	41	11.1^c^
MI	75	369	392	2.3^c^	198	218	10	1.4^c^	106	1,015	858	4.3^c^	26	69	165	13.0^c^
MN	9	45	400	3.5	168	169	1	3.2^c^	12	127	958	5.2^c^	11	81	636	15.3^c^
MO	134	226	69	1.2	301	333	11	1.2^c^	311	585	88	1.3^c^	29	50	72	5.5^c^
ND	35	44	26	−1.6	105	454	332	3.5^c^	44	123	180	2.4^c^	11	17	55	7.1^c^
NE	101	322	219	2.1	307	371	21	1.9^c^	326	896	175	2.1^c^	150	85	−43	−3.4
OH	172	458	166	1.3	279	303	9	1.4^c^	227	1,259	455	3.1^c^	41	80	95	14.0^c^
SD	21	254	1,110	5.1^c^	131	442	237	3.7^c^	38	1,641	4,218	6.8^c^	12	15	25	6.6^c^
WI	136	173	27	−0.1	170	336	98	1.7^c^	197	870	342	2.6^c^	44	97	120	14.0^c^
**South**																
AL	458	326	−29	−0.6	753	489	−35	−0.1	514	1,098	114	1.5^c^	116	125	8	1.1^c^
AR	135	269	99	2.0	436	464	6	0.8	313	796	154	1.6^c^	57	58	2	2.1
DC	13	50	285	2.2^c^	76	144	89	2.9^c^	30	133	343	3.0^c^	8	23	188	11.0^c^
DE	35	200	471	2.0^c^	239	333	39	1.3^c^	102	662	549	3.9^c^	25	63	152	10.5^c^
FL	90	335	272	2.4^c^	591	634	7	0.9^c^	160	809	406	3.4^c^	58	99	71	22.4^c^
GA	242	334	38	0.4	460	374	−19	0.5	544	832	53	0.7^c^	92	92	0	0
KY	234	445	90	0.5	234	254	9	0.4	489	945	93	1.3^c^	53	120	126	5.5^c^
LA	106	182	72	0.6	525	238	−55	−0.8	229	555	142	1.4^c^	29	45	55	7.1^c^
MD	38	227	497	3.2^c^	224	346	54	1.4	111	742	568	3.6^c^	41	67	63	20.2^c^
MS	303	229	−24	−0.3	729	389	−47	−0.5	825	680	−18	−0.1	44	181	311	11.3^c^
NC	136	254	87	1.4	564	334	−41	0	243	863	255	2.6^c^	79	66	−16	2.7^c^
OK	117	184	57	0.9	274	220	−20	0.1	330	552	67	0.9^c^	40	63	58	6.8^c^
SC	179	327	83	0.9	804	321	−60	−0.3	497	875	76	0.6	133	116	−13	3.6^c^
TN	235	207	−12	−0.2	509	324	−36	−0.3	569	583	2	0.7	44	87	98	5.9^c^
TX	70	242	246	1.8	328	271	−17	−0.3	161	495	207	2.2^c^	25	56	124	13.7^c^
VA	108	282	161	1.4	409	346	−15	0.6	407	724	78	1.1^c^	96	138	44	14.0^c^
WV	298	530	78	1.2	276	785	184	0.9	527	1,173	123	1.7^c^	211	258	22	13.9^c^
**West**																
AZ	77	205	166	1.7^c^	230	384	67	1.1^c^	145	454	213	2.2^c^	75	96	28	6.2^c^
CA	35	151	331	2.2^c^	168	308	83	1.7^c^	49	431	780	4.0^c^	14	42	200	13.3^c^
CO	57	319	460	3.0^c^	235	224	−5	0.8	115	1,038	803	3.9^c^	57	82	44	5.5^c^
ID	90	344	282	2.6^c^	191	287	50	0.5	344	691	101	2.1^c^	50	80	60	10.6^c^
MT	32	112	250	2.4	231	327	42	4.3^c^	144	255	77	2.8^c^	18	62	244	8.3^c^
NM	104	133	28	0.4	242	237	−2	0.3	169	252	49	1.3^c^	88	92	5	0.2
NV	61	349	472	2.3^c^	512	774	51	0.4	115	1,030	796	2.8^c^	76	131	72	7.5^c^
OR	73	246	237	1.9^c^	273	328	20	0	95	699	636	3.6^c^	45	93	107	8.8^c^
UT	188	370	97	1	214	287	34	−1.2	895	1,229	37	0.8	85	203	139	15.7^c^
WA	35	248	609	2.4^c^	269	326	21	1	118	677	474	3.5^c^	45	71	58	9.4^c^
WY	45	44	−2	−0.3	92	93	1	0.4	158	147	−7	0.1	11	20	82	5.6^c^

Abbreviation: AMPC, average monthly percentage change.

a Average monthly unit sales. Equivalent sales per 100,000 people; 1 unit is equal to 1 rechargeable or 1 disposable e-cigarette, 1 pack of 5 prefilled cartridges, or 1 bottle of e-liquid.

b AMPC is average percentage change per 4-week period. For rechargeables, disposables, and prefilled cartridges, calculations included all periods from 2012 through 2016 (n = 65). For e-liquids, calculations included all periods from 2014 through 2016 with non-zero sales.

c Indicates significant change (α = .05).

d No e-liquid sales were reported in the data until mid-2013. Percentage change reflects change from 2014 to 2016.

**Figure 1:**
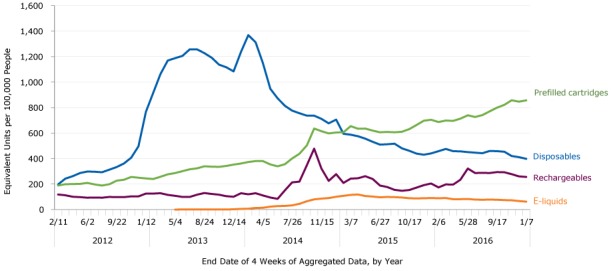
Unit sales of e-cigarettes by product type and by 4-week periods, United States 2012–2016. One unit is equal to 1 rechargeable, 1 disposable, 5 prefilled cartridges, or 1 e-liquid bottle. **Table:** 

Despite overall sales growth, sales fluctuated across time by product type (Figure 1). Specifically, sales of rechargeables, prefilled cartridges, and e-liquids grew relatively steadily over time, while sales for disposables were less consistent. In the final 4 weeks of the study period, prefilled cartridge sales rate peaked at more than 856 units. In contrast, sales of disposables increased sharply in late 2012 before peaking in 2013 as the product with the highest sales rate. Disposables later decreased in sales from 2014 to 2016.

End Date	Rechargeables	Disposables	Prefilled Cartridges	E-Liquids
**2012**				
2/11	117.2	197.3	188.6	—
3/10	112.7	242.7	198.2	—
4/7	100.3	261.2	199.4	—
5/5	97.1	286.4	201.4	—
6/2	93.0	298.8	208.1	—
6/30	93.9	297.0	197.6	—
7/28	93.4	292.3	189.0	—
8/25	98.8	312.8	198.1	—
9/22	96.9	332.7	226.5	—
10/20	97.8	361.3	234.1	—
11/17	102.6	407.1	255.2	—
12/15	103.3	495.5	250.3	—
1/12	125.5	767.9	244.5	—
**2013**				
2/9	125.1	915.3	239.6	—
3/9	127.6	1063.6	256.8	—
4/6	115.6	1168.6	276.2	—
5/4	107.6	1187.7	286.8	0
6/1	98.4	1205.0	300.6	0
6/29	98.8	1257.3	315.2	0
7/27	116.7	1257.1	322.5	0
8/24	129.7	1228.1	338.9	0
9/21	121.9	1191.6	335.8	0.1
10/19	116.9	1136.1	334.9	0.1
11/16	105.0	1116.2	342.4	0.7
12/14	100.3	1083.6	352.0	2.7
1/11	128.4	1237.9	361.2	4.8
**2014**				
2/8	119.8	1368.3	373.1	6.7
3/8	128.2	1312.7	380.8	12.6
4/5	110.8	1149.5	379.7	14.4
5/3	94.2	948.2	354.6	22.2
5/31	83.9	872.5	338.6	26.9
6/28	150.8	813.6	355.4	29.1
7/26	213.7	776.0	401.1	33.3
8/23	218.8	755.4	437.2	45.3
9/20	348.7	736.3	503.1	65.7
10/18	477.6	736.9	634.9	79.8
11/15	322.2	711.2	614.5	86.0
12/13	224.3	676.1	596.8	89.6
1/10	277.5	706.0	604.3	100.7
**2015**				
2/7	208.4	594.7	605.4	108.1
3/7	242.8	586.7	653.8	115.5
4/4	245.7	575.4	634.6	118.0
5/2	260.9	556.3	635.7	106.6
5/30	239.9	531.0	620.0	101.8
6/27	188.7	508.9	605.8	96.2
7/25	176.2	512.2	609.4	98.6
8/22	155.3	517.3	606.5	97.2
9/19	147.0	479.6	610.4	93.8
10/17	152.9	460.5	631.2	88.3
11/14	171.9	439.7	663.7	86.5
12/12	191.3	429.7	696.5	88.2
1/9	204.6	440.0	704.9	90.8
**2016**				
2/6	173.0	457.3	686.3	88.1
3/5	197.0	474.9	698.7	89.6
4/2	195.9	458.7	695.3	81.5
4/30	234.0	456.7	713.7	81.6
5/28	322.1	450.6	739.8	82.5
6/25	286.4	446.1	727.0	78.4
7/23	288.5	442.6	741.1	76.7
8/20	286.1	459.7	770.1	77.7
9/17	294.5	457.9	799.5	76.3
10/15	292.6	453.5	821.0	73.3
11/12	277.3	419.2	857.3	72.6
12/10	260.2	411.5	846.6	66.1
1/7	256.3	398.3	856.3	61.9

### State sales trends

Each of the 48 states and Washington, DC, had significant average monthly sales growth for at least 1 e-cigarette product type (Table 1). Ten states (Maine, New Hampshire, New Jersey, New York, Michigan, South Dakota, Delaware, Florida, Arizona, California) and Washington, DC, had significant average monthly sales growth for all 4 product types. Between 2012 and 2016, the most prominent increases were observed in South Dakota, where scanner data indicated a relative percentage increase of 1,110% for rechargeables (AMPC = 5.1) and 4,218% for prefilled cartridges (AMPC = 6.8); and in Illinois, where data indicated a relative percentage increase of 430% for disposables (AMPC = 3.6). Between 2014 and 2016, Minnesota had the highest relative increase for e-liquid sales at 636% (AMPC = 15.3). In 2016, the highest average monthly sales rate occurred in New Hampshire for rechargeables (622 units), Illinois for disposables (1,527 units), South Dakota for prefilled cartridges (1,641 units), and West Virginia for e-liquids (258 units).

### National price trends

Nationally, average e-cigarette prices decreased by 48% for rechargeables (AMPC = −1.2), 14% for disposables (AMPC = −0.2), and 12% for prefilled cartridges (AMPC = −0.4) from 2012 through 2016 (Table 2). E-liquid prices, however, did not significantly change across the study period. In 2016, the average e-cigarette price was highest for a unit of prefilled cartridges ($14.36), followed by rechargeables ($10.33), disposables ($8.01), and e-liquids ($6.83).

**Table 2:** Average E-Cigarette Prices by Product Type, United States, 2012–2016

State	Rechargeables				Disposables				Prefilled Cartridges				E-Liquids			
	Average Price, $^a^		Change, %	AMPC^b^	Average Price, $^a^		Change, %	AMPC^b^	Average Price, $^a^		Change, %	AMPC^b^	Average Price, $^a^		Change, %^d^	AMPC^b^
	2012	2016			2012	2016			2012	2016			2014	2016		
**All**	19.81	10.33	−48	−1.2^c^	9.29	8.01	−14	−0.2^c^	16.37	14.36	−12	−0.4^c^	7.51	6.83	−9	−0.1
**Northeast**																
CT	26.23	12.51	−52	−1.3^c^	10.01	9.52	−5	−0.1	15.89	18.04	14	0.1	7.72	7.45	−3	−0.1
MA	23.78	10.45	−56	−1.5^c^	10.12	9.39	−7	−0.1^c^	16.29	16.82	3	−0.2	7.88	6.38	−19	−0.9^c^
ME	18.36	10.14	−45	−1.2^c^	10.22	9.20	−10	−0.4^c^	14.91	15.17	2	−0.2	7.60	6.52	−14	−1.1
NH	18.79	9.74	−48	−1.4^c^	9.84	8.68	−12	−0.3^c^	15.05	16.61	10	0.1	7.38	7.40	0	0
NJ	28.00	9.15	−67	−1.8^c^	9.80	8.95	−9	−0.1	18.95	16.62	−12	−0.4^c^	7.69	6.94	−10	0.3
NY	23.12	10.15	−56	−1.7^c^	9.51	9.16	−4	0	19.05	15.02	−21	−0.9^c^	7.60	6.40	−16	−0.1
PA	23.76	11.31	−52	−0.7	9.04	8.70	−4	0.3^c^	16.63	13.28	−20	−0.4^c^	7.54	6.90	-8	0
RI	23.23	11.30	−51	−1.1^c^	9.50	9.22	−3	0	17.82	15.96	−10	−0.8^c^	8.10	7.18	−11	−1.0^c^
VT	13.91	12.01	−14	−1.0^c^	9.28	9.09	−2	−0.3	15.35	15.17	−1	−0.3	7.22	5.54	−23	−0.5
**Midwest**																
IA	26.64	12.15	−54	−1.5^c^	9.37	8.43	−10	−0.2^c^	16.90	15.03	−11	−0.2^c^	7.60	7.98	5	0.5
IL	20.29	11.82	−42	−0.4	9.85	6.20	−37	−0.8^c^	16.36	14.72	−10	−0.3^c^	8.05	7.06	−12	−0.5^c^
IN	18.16	9.78	−46	−1.0^c^	9.60	8.12	−15	−0.3^c^	15.84	15.18	−4	0	8.24	7.97	−3	0.3
KS	25.44	8.93	−65	−1.7^c^	9.27	8.26	−11	−0.2	16.93	15.81	−7	0	7.53	6.85	−9	0.3
MI	17.32	9.78	-44	−1.1^c^	9.09	7.37	−19	−0.4^c^	15.67	11.87	−24	−0.6^c^	7.36	6.45	−12	−0.1
MN	22.35	15.00	−33	−0.7	10.97	11.20	2	0.2	23.14	17.12	−26	0.4	7.16	7.91	10	0.5
MO	21.58	12.12	−44	−0.7	9.28	8.17	−12	−0.2^c^	15.95	13.58	−15	−0.2^c^	7.93	7.45	−6	−0.3
ND	28.21	12.71	−55	−1.1	8.88	7.99	−10	0.1	16.76	13.23	−21	−0.6^c^	7.24	7.39	2	0.6
NE	22.23	12.13	−45	−0.7	9.24	8.20	−11	−0.2^c^	15.40	12.83	−17	−0.3	7.95	7.09	−11	0.1
OH	17.56	9.81	−44	−1.0^c^	9.05	8.08	−11	−0.4^c^	16.05	13.94	−13	−0.2	7.01	7.51	7	−0.1
SD	31.85	12.06	−62	−1.4^c^	8.97	8.03	−10	−0.3^c^	16.12	15.56	−3	0.2	7.19	8.44	17	1.4
WI	17.62	12.83	−27	−0.4	9.82	7.58	−23	−0.4^c^	15.84	16.39	3	0.2^c^	6.90	7.30	6	0.4
**South**																
AL	15.90	10.99	−31	−0.8^c^	9.43	7.49	−21	−0.4^c^	16.31	12.18	−25	−0.5^c^	6.65	5.32	−20	−0.4
AR	18.06	11.93	−34	−1.1^c^	8.86	7.95	−10	−0.1	15.91	13.49	−15	0.1	6.03	6.68	11	0.4^c^
DC	28.20	18.43	−35	−0.5	9.64	14.75	53	0.7^c^	18.85	20.58	9	−0.2	7.88	10.19	29	1.6^c^
DE	27.80	10.17	−63	−1.7^c^	9.93	8.22	−17	−0.4^c^	15.73	16.04	2	0	6.78	6.95	3	0.2
FL	19.29	9.77	−49	−1.2^c^	9.40	8.25	−12	−0.3^c^	19.13	14.38	−25	−0.8^c^	7.60	7.31	−4	0.8^c^
GA	17.47	10.43	−40	−1.1^c^	9.03	8.53	−6	−0.1^c^	15.87	14.54	−8	−0.1	7.62	6.92	−9	−0.4^c^
KY	17.81	9.35	−47	−1.5^c^	9.39	8.12	−14	−0.2^c^	15.26	13.96	−9	0.1	7.93	7.36	−7	−0.6^c^
LA	22.36	10.98	−51	−1.3^c^	9.41	8.04	−15	−0.3^c^	16.19	11.90	−26	−0.5^c^	6.00	7.95	33	3.3^c^
MD	25.65	9.55	−63	−1.7^c^	8.92	7.87	−12	−0.1	17.60	15.14	−14	−0.5^c^	8.38	6.67	−20	−0.9
MS	19.51	10.76	−45	−1.4^c^	9.13	7.81	−14	−0.3^c^	16.07	13.36	−17	−0.2	7.93	5.70	−28	−1.7^c^
NC	19.02	10.87	−43	−1.1^c^	9.06	7.58	−16	−0.2	15.88	12.84	−19	−0.3	7.35	6.90	−6	−0.1
OK	19.39	11.78	−39	−1.1^c^	9.20	8.14	−12	−0.2^c^	14.91	10.43	−30	−0.6^c^	7.36	6.78	−8	0.2
SC	19.31	10.70	−45	−1.3^c^	8.90	8.33	−6	−0.2	15.99	12.43	−22	−0.6^c^	6.60	6.48	−2	0
TN	19.16	11.96	−38	−1.0^c^	9.16	7.63	−17	−0.4^c^	15.69	13.80	−12	0	7.74	6.00	−22	−0.8^c^
TX	19.76	8.73	−56	−1.5^c^	9.20	7.97	−13	−0.2^c^	16.67	12.20	−27	−0.2	7.82	6.84	−13	0.7^c^
VA	22.46	8.87	−61	−1.7^c^	8.73	7.90	−10	−0.3^c^	17.37	12.88	−26	−0.7^c^	7.33	6.47	−12	1.1
WV	15.88	8.38	−47	−1.7^c^	9.12	6.51	−29	−0.3	15.89	11.39	−28	−0.6^c^	6.94	6.34	−9	−0.9
**West**																
AZ	21.99	11.62	−47	−1.0^c^	9.59	7.64	−20	−0.4^c^	16.08	15.08	−6	0.1	8.00	7.26	−9	−0.4^c^
CA	23.26	11.78	−49	−0.6^c^	8.99	8.05	−10	−0.3^c^	17.53	13.97	−20	−0.4^c^	7.43	6.98	−6	0.1
CO	26.58	10.10	−62	−1.5^c^	9.38	8.20	−13	−0.2^c^	15.74	12.68	−19	−0.7^c^	7.64	7.22	−5	0.2
ID	22.17	9.17	−59	−1.8^c^	9.17	7.71	−16	−0.4^c^	15.43	16.09	4	0.3	6.89	6.29	−9	0.1
MT	25.94	11.54	−56	−1.3	8.87	6.82	−23	−0.6^c^	15.70	13.83	−12	0.3	6.14	5.34	−13	0
NM	19.70	10.53	−47	−1.3^c^	9.61	7.84	−18	−0.3^c^	15.24	15.42	1	0.2	7.96	7.07	−11	0
NV	24.38	11.93	−51	−1.0^c^	8.83	7.60	−14	−0.1	16.44	14.77	−10	0	7.72	6.96	−10	−0.4
OR	23.14	11.09	−52	−0.6	9.07	7.89	−13	−0.1	15.72	15.43	−2	−0.1	7.42	6.36	−14	0.4
UT	20.69	9.43	−54	−1.5^c^	8.70	6.70	−23	−0.4^c^	14.34	13.00	−9	−0.2^c^	7.63	6.04	−21	−0.5
WA	25.48	10.04	−61	−1.3^c^	8.86	7.81	−12	−0.2	16.05	14.88	−7	0	7.19	6.67	−7	0.5
WY	28.40	13.21	−53	−1.2^c^	9.51	8.19	−14	−0.3^c^	15.01	15.76	5	0.2^c^	7.34	8.16	11	1.2^c^

a Average price per unit; 1 unit is equal to 1 rechargeable or 1 disposable e-cigarette, 1 pack of 5 prefilled cartridges, or 1 bottle of e-liquid.

b AMPC is the average monthly percentage change, or the average percentage change per 4-week period. For rechargeables, disposables, and prefilled cartridges, calculations included all periods from 2012 through 2016 (n = 65). For e-liquids, calculations included all periods from 2014 through 2016 with non-zero sales.

c Indicates significant change (α = .05).

d No e-liquid sales were reported in the data until mid-2013. Percentage change reflects change from 2014 to 2016.

**Figure 2:**
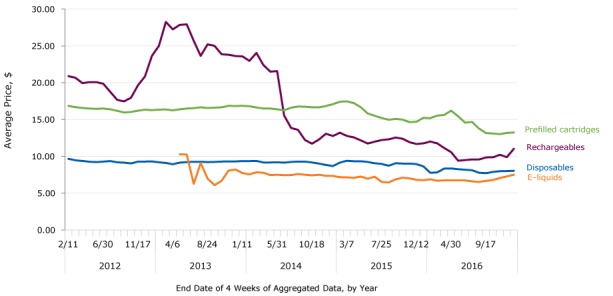
Average prices of e-cigarettes by product type and by 4-week periods, United States 2012–2016. Average prices were calculated by using inflation-adjusted dollars (2016) and raw unit sales. Only items sold in the most common package size for each product were included in average price calculations. **Table:** 

Similar to national trends in unit sales, national trends in e-cigarette prices fluctuated over time (Figure 2). Rechargeable e-cigarette prices fluctuated during the beginning of the study period, peaking in early 2013 around $27. Prices then decreased by more than 55% to approximately $12 in October 2014, before decreasing more steadily throughout 2015 and 2016. Despite fluctuations shortly after they first appeared in convenience stores and all other outlets combined, e-liquid prices averaged approximately $7 from late 2013 through the end of the study period.

End Date	Rechargeables, $	Disposables, $	Prefilled Cartridges, $	E-Liquids, $
**2012**				
2/11	20.90	9.65	16.86	—
3/10	20.67	9.45	16.68	—
4/7	19.94	9.36	16.56	—
5/5	20.07	9.25	16.49	—
6/2	20.08	9.20	16.45	—
6/30	19.85	9.28	16.49	—
7/28	18.75	9.35	16.38	—
8/25	17.67	9.19	16.17	—
9/22	17.47	9.15	15.96	—
10/20	17.96	9.03	16.01	—
11/17	19.64	9.27	16.20	—
12/15	20.85	9.28	16.33	—
1/12/13	23.64	9.30	16.26	—
**2013**				
2/9	25.00	9.19	16.34	—
3/9	28.25	9.09	16.36	—
4/6	27.25	8.91	16.25	—
5/4	27.85	9.15	16.38	10.27
6/1	27.92	9.22	16.50	10.25
6/29	25.69	9.26	16.54	6.26
7/27	23.64	9.26	16.66	9.10
8/24	25.22	9.21	16.56	6.95
9/21	25.00	9.22	16.61	6.09
10/19	23.87	9.27	16.65	6.67
11/16	23.81	9.30	16.86	8.05
12/14	23.61	9.31	16.83	8.19
1/11/14	23.56	9.35	16.85	7.71
**2014**				
2/8	22.97	9.34	16.80	7.55
3/8	24.04	9.36	16.63	7.85
4/5	22.39	9.17	16.50	7.77
5/3	21.50	9.16	16.50	7.45
5/31	21.56	9.19	16.39	7.50
6/28	15.55	9.14	16.24	7.43
7/26	13.85	9.24	16.60	7.44
8/23	13.58	9.27	16.77	7.59
9/20	12.21	9.27	16.74	7.50
10/18	11.72	9.16	16.64	7.42
11/15	12.28	9.01	16.64	7.50
12/13	13.07	8.84	16.83	7.35
1/10/15	12.74	8.67	17.07	7.33
**2015**				
2/7	13.19	9.14	17.42	7.16
3/7	12.77	9.39	17.46	7.15
4/4	12.56	9.32	17.23	7.08
5/2	12.18	9.32	16.68	7.26
5/30	11.73	9.23	15.81	6.96
6/27	11.97	9.06	15.50	7.22
7/25	12.21	8.96	15.20	6.53
8/22	12.31	8.72	14.95	6.43
9/19	12.55	9.06	15.07	6.86
10/17	12.40	9.02	14.97	7.09
11/14	11.93	9.01	14.65	7.00
12/12	11.67	8.94	14.68	6.80
1/9/16	11.76	8.62	15.23	6.76
**2016**				
2/6	12.02	7.77	15.18	6.87
3/5	11.75	7.82	15.52	6.68
4/2	11.12	8.34	15.61	6.72
4/30	10.56	8.36	16.19	6.73
5/28	9.41	8.24	15.43	6.73
6/25	9.47	8.18	14.58	6.73
7/23	9.58	8.10	14.67	6.60
8/20	9.57	7.78	13.78	6.50
9/17	9.85	7.70	13.16	6.65
10/15	9.87	7.88	13.09	6.77
11/12	10.21	7.98	13.01	7.05
12/10	9.89	8.00	13.18	7.27
1/7/2017	11.01	8.04	13.24	7.50

### State price trends

From 2012 through 2016, average monthly e-cigarette prices significantly decreased in 39 states for rechargeables, 31 states for disposables, 20 states for prefilled cartridges, and 8 states for e-liquids. In contrast, we found significant average monthly price increases in 1 state (Pennsylvania) and Washington, DC, for disposables, 2 states (Wyoming and Wisconsin) for prefilled cartridges, and 5 states (Arkansas, Florida, Louisiana, Texas, Wyoming) and Washington, DC, for e-liquids.

In 2016, West Virginia had the lowest average monthly sales price for rechargeables ($8.38), Illinois for disposables ($6.20), Oklahoma for prefilled cartridges ($10.43), and Alabama for e-liquids ($5.32). The highest average unit prices for each product type in 2016 were in Washington, DC, where monthly average prices per unit were $18.43 for rechargeables, $14.75 for disposables, $20.58 for prefilled cartridges, and $10.19 for e-liquids.

## Discussion

From 2012 through 2016, e-cigarette unit sales in the United States significantly increased for all assessed product types, including rechargeables, disposables, prefilled cartridges, and e-liquids. At the state level, monthly unit sales significantly increased for at least 1 product type in all 48 states and Washington, DC. During the same period, national e-cigarette prices significantly decreased for all product types with the exception of e-liquids, which increased in price in 5 states and Washington, DC. Taken together, these findings underscore the rapidly evolving landscape of the US e-cigarette retail marketplace, with decreases in unit price accompanying increases in sales trends overall. Furthermore, prominent shifts occurred by product type, with prefilled cartridges having the highest average sales and the lowest average price in 2016.

Variations in US e-cigarette sales and prices have persisted since the first national and state assessment of their retail sales at convenience stores and all other outlets combined; during 2012–2013, the revenue of e-cigarettes in those retail channels increased by 320% for disposables, 72% for starter kits, and 82% for prefilled cartridges (13). Our study, however, found that disposable products no longer dominate e-cigarette sales in these retail channels: rechargeables had the largest relative percentage increase in sales from 2012 through 2016. Furthermore, prefilled cartridge sales increased substantially by more than 256% through 2016, surpassing all other product types to become the most commonly sold unit of e-cigarette products in convenience stores and all other outlets combined. Additionally, e-liquids had rapid sales growth, with an average monthly percentage increase of 5.9% in national unit sales during 2014–2016. These findings are generally consistent with industry reports indicating that disposables comprise a declining proportion of e-cigarette retail sales (9,17). This decline may reflect an underlying shift in product preference among consumers; established e-cigarette users who are current or former smokers, for instance, can graduate to using more customizable devices featuring open systems (1,18).

Price gaps for e-cigarette devices have narrowed over time. During the 4-week period ending on June 29, 2013, the national average price of rechargeables ($25.69) was more than 3 times the average price of disposables ($9.26). Rechargeable prices have since plummeted; in 2016, the average price difference between a rechargeable unit and a disposable unit was less than $2. In contrast, although the average price of both prefilled cartridges and e-liquids decreased, a unit of prefilled cartridges was approximately twice the average price of a bottle of e-liquid throughout the study period. Overall, the increase in e-cigarette sales and decrease in price is consistent with previous studies demonstrating that e-cigarette sales are responsive to their own price changes (19). These trends suggest that, if e-cigarette prices continue to decrease, their sales may also continue to rise. However, other factors, such as the pricing and consumption of other conventional or newer tobacco products, will affect e-cigarette demand (1).

Sales data at the federal and subnational levels can help inform and evaluate efforts to regulate e-cigarettes at the national, state, and local levels. In 2009, the Family Smoking Prevention and Tobacco Control Act (20) gave the US Food and Drug Administration (FDA) the authority to regulate the manufacturing, distribution, and marketing of cigarettes, roll-your-own cigarette tobacco, and smokeless tobacco sold in the United States. In May 2016, the FDA subsequently issued a deeming rule to extend its authority over all tobacco products, including e-cigarettes and their components and parts (eg, cartridges) (21).

The Family Smoking Prevention and Tobacco Control Act preserves state and local authority over implementing certain regulations in addition to some actions not otherwise covered by the Act that are expressly under the purview of state and local authority. These actions include restricting tobacco use in public places, levying taxes on tobacco products, raising the age of sale above 18, and restricting sales by certain retailers (1,21). With regard to e-cigarettes, some states and localities have implemented strategies to both minimize the potential harms of e-cigarette consumption at the population level, particularly among youth and young adults, and to maximize any potential benefits for current adult smokers (1,2). As of June 2017, 46 states and Washington, DC, have minimum legal age restrictions on the purchase of e-cigarettes; 15 states require a retail license to sell e-cigarettes over the counter; 8 states (California, Delaware, Hawaii, New Jersey, North Dakota, Oregon, Utah, Vermont), Washington, DC, and Puerto Rico have comprehensive smoke-free indoor air laws that prohibit smoking and using e-cigarettes in indoor areas of private worksites, restaurants, and bars; and 7 states (California, Kansas, Louisiana, Minnesota, North Carolina, Pennsylvania, West Virginia) and Washington, DC, have enacted e-cigarette taxation policies (22,23). Although the long-term impact of these population-based strategies on e-cigarette sales in US retail outlets continues to be assessed, cross-sectional audits from nationally representative samples of tobacco retailers suggest that e-cigarettes are more likely to be available in retail outlets in areas with lower e-cigarette prices and less comprehensive smoke-free air policies (24). Moreover, the US Surgeon General indicated that higher prices and comprehensive smoke-free air policies are among the most effective methods to prevent initial use of conventional tobacco products among adolescents and young adults (2).

Our study indicates that the average price across all e-cigarette types in 2016 was highest in Washington, DC, which may be attributable, in part, to the city’s taxation of e-cigarettes at 67% of the wholesale price (25). In contrast, states with the lowest average prices for rechargeables (West Virginia), disposables (Illinois), prefilled cartridges (Oklahoma), and e-liquids (Alabama) do not currently have state e-cigarette tax policies. Previous research suggests that a 10% increase in the price of e-cigarettes would reduce the sales of disposable and rechargeable e-cigarettes by approximately 12% and 19%, respectively (19). Thus, the continued monitoring of e-cigarette sales data in the context of these evolving strategies may inform the implementation and sustainment of related tobacco control policies and practices.

Our study has several limitations. First, Nielsen’s projection methods are proprietary. However, Nielsen scanner data are widely used in academic and marketing research, and previous sales estimates are consistent with US Treasury and securities analyst reports (10,17,26). Second, these data are derived from product sales at traditional tobacco retail outlets and do not include sales from tobacco specialty or vape shops or the internet because data for these venues are not commercially available (1); these omitted outlets were estimated to account for a majority of the overall electronic nicotine delivery system market in 2017 (17). Third, our study may not have captured the full range of data on e-cigarette products currently available on the US market given that more advanced products, such as tank-style systems, are predominantly sold in vape shops and on the internet (27,28). Finally, our study could not account for coupons or promotions applied at the point of sale.

From 2012 through 2016, e-cigarette unit sales in the United States generally increased as product prices decreased. Moreover, notable shifts in sales occurred by product type; prefilled cartridges, in particular, had the highest average unit sales and the highest average unit price in 2016. Given that e-cigarettes have a range of potential impacts on individual and population health (2), ongoing surveillance of e-cigarette sales is important to help inform tobacco control policies and practices. Strategies at the national, state, and local level, including proven population-based tobacco interventions (1,2,29), will be critical to minimize harms and maximize any potential benefits that e-cigarettes could have on individual and population-level health (1,2).
